# Fear of nuclear war increases the risk of common mental disorders among young adults: a five-year follow-up study

**DOI:** 10.1186/1471-2458-4-42

**Published:** 2004-09-30

**Authors:** Kari Poikolainen, Terhi Aalto-Setälä, Annamari Tuulio-Henriksson, Mauri Marttunen, Jouko Lönnqvist

**Affiliations:** 1 Finnish Foundation for Alcohol Studies, PO Box 220, FIN-00531 Helsinki, Finland; 2 National Public Health Institute, Department of Mental Health and Alcohol Research, Mannerheimintie 166, FIN-00300 Helsinki, Finland; 3 Helsinki University Hospital, Hospital for Children and Adolescents, Department of Child Psychiatry, Helsinki, Finland; 4 Helsinki University Hospital, Department of Adolescent Psychiatry, Peijas Hospital, FIN-01400 Vantaa, Finland; 5 Tampere School of Public Health, University of Tampere, FIN-33014 Finland; 6 Department of Psychiatry, University of Helsinki, Helsinki, Finland

## Abstract

**Background:**

Evidence on the relation between fear of war and mental health is insufficient. We carried out a prospective cohort study to find out whether fear of nuclear war is related to increased risk of common mental disorders.

**Methods:**

Within two months preceding the outbreak of Persian Gulf War in January 1991, 1518 adolescents [mean age 16.8 years, SD 0.9] filled in a self-administered questionnaire. Of the 1493 respondents, 47% gave their written informed consent to participate in the follow-up study. There were no material differences between those who chose to respond anonymously and those who volunteered to give their name and address for the follow-up study. In 1995, the response to the follow-up questionnaire was 92%. Common mental disorders were assessed by 36-item version of the General Health Questionnaire [GHQ]. A score 5 or higher was considered to indicate caseness. We excluded 23 cases which had used mental health services in the year 1991 or earlier and two cases with deficient responses to GHQ. This left 626 subjects for analysis [400 women].

**Results:**

After adjusting for significant mental health risk factors in logistic regression analysis, the risk for common mental disorders was found to be significantly related to the increasing frequency of fear for nuclear war, high scores of trait anxiety and high scores of immature defense style. Elevated risk was confined to the group reporting fear of nuclear war once a week or more often [odds ratio 2.05; 95% confidence interval 1.29–3.27].

**Conclusion:**

Frequent fear of nuclear war in adolescents seems to be an indicator for an increased risk for common mental disorders and deserves serious attention.

## Background

Risks of war and terrorism are threatening our health, both directly in actual life and also indirectly by the increasingly violent content of video games and other forms of entertainment. How does this affect mental health? Earlier during the cold war period, fear of war was found to be common among adolescents, and more prevalent among girls than boys [1-5]. Little is known about the influence of fear of war on mental health of adolescents. On one hand, it has been argued that worrying about nuclear war is related to positive aspects of mental health [6]. On the other, fear of nuclear war has been found to associate with several measures of psychological distress in cross-sectional studies [4,7-9]. To our knowledge, no follow-up studies have been published. However, high perceived risk of nuclear war might be related not only to transient psychological distress but also to more long-term mental disorder among vulnerable adolescents. We have followed up a cohort of adolescents first studied during the period of increasing international tension before the outbreak of the Persian Gulf War in January 17, 1991, and report here on the relation between fear of nuclear war at that time and incident common mental disorders five years later.

## Methods

### Design

Between December 4, 1990, and January 16, 1991, 1518 adolescents from five high schools in Helsinki and five in Jyväskylä, Finland, representing a cross-section of school entrance requirement levels filled in a self-administered questionnaire during an ordinary classroom hour. Of the 1493 respondents, 709 (47%) gave their written informed consent to participate in the follow-up study. There were no significant differences between those who chose to respond anonymously and those who volunteered to give their name and address for the follow-up study with respect to baseline predominance of mature, immature or neurotic defence styles, trait anxiety, trait depression, the number of positive and that of negative life-events, self-esteem, coherence of future, or availability of social support. Anonymous respondents reported less somatic symptoms than those who gave informed consent to follow up. The absolute difference in the symptom score was not very large, however. The mean scores (SE) were 21.8 (0.22) and 22.8 (0.29) for men, 24.2 (0.23) and 25.0 (0.23) for females, respectively [10]. Of the 709 subjects who gave signed consent, two were excluded from the follow-up due to deficient completion of the baseline questionnaire, and one died. The sample eligible for follow-up comprised 706 subjects. In 1995, the response to the follow-up questionnaire was 92%. Design and sample has been described earlier in more detail [10].

### Participants

We excluded 23 cases who reported having used mental health services in the year 1991 or earlier and two cases with deficient responses to GHQ. This left 626 subjects for analysis, of whom 400 were women. At the baseline, the age range was 15 to 19 years (mean 16.8, SD 0.9).

### Measures

#### Baseline examination in 1990

Frequency of fearing nuclear war during past four weeks (scores in parentheses) was assessed by a question with six options: not at all (0), less than once a week (0.5), 1–2 times a week (6), 3–5 times a week (16), almost daily (22) and daily (28).

The Defence Style Questionnaire (DSQ) consisted of 72 statements assessing possible conscious derivatives of 20 defences. It is based on the 88-item version of the Bond's Defense Style Questionnaire [11]. Andrews et al. [12] reviewed the items to make the labelling consistent with the Diagnostic and Statistical Manual of Mental Disorders (3rd ed. revised, DSM-III-R) by the American Psychiatric Association [13]. The defence styles were grouped into three levels: mature, neurotic, and immature defence styles. Individual defences are (mature:) sublimation, humour, anticipation, suppression, (neurotic:) undoing, altruism, idealisation, reaction formation, (immature:) projection, passive aggression, acting out, isolation, devaluation, autistic fantasy, denial, displacement, dissociation, splitting, rationalisation and somatization [14].

The Trait Anxiety Inventory was used to measure trait anxiety as a general tendency of feeling [15]. Trait anxiety is used to screen neurotic anxiety problems and vulnerability for anxiety disorders.

Depressive trait [16] was assessed by questions following the style, scoring and response options of the Trait Anxiety Inventory. The questions dealt with a general tendency to have obvious depressive mood.

An abbreviated version of the Life Event Checklist [17] consisted of 20 defined life events considered to be the most common ones among Finnish adolescents and of four open items. Number of negative and that of positive life events was analysed.

The Somatic Symptom Score is an abbreviated 14-item version of an original 18-item score used earlier in Finnish studies on adults and adolescents [18]. The 14 items comprised physical symptoms common in adolescence but only rarely associated with a physical disease, such as headache, abdominal pains, fatigue or weakness, lack of energy, diarrhoea or irregular bowel function. Respondents were asked "Have any of the following symptoms bothered you, and how often during the last six months?" The response options were never, sometimes, quite often, and often or continuously.

The self-esteem scale by Rosenberg [19] consists of ten items measuring the self acceptance aspect of self-esteem. Rosenberg relates positive self-esteem to many social and interpersonal consequences such as less shyness and depression, more assertiveness, and more extra-curricular activities. The response scorings were inverted so that a high total score indicated high self-esteem.

Coherence of future was measured by three items (no. 11, 22 and 27) from the Sense of Coherence Scale [20] relating to the meaningfulness and manageability of one's own personal future.

Social support was ascertained by asking "Do you have a significant other person with whom you may discuss your personal activities and problems?".

Social class assessment was based on father's occupation or on mother's occupation when the father was not living in the family of the adolescent. Use of the City of Helsinki Social Group Classification divided the sample into four categories: (i) professionals, managers and higher administrative or clerical employees, (ii) lower clerical employees, (iii) skilled workers, and (iv) unskilled workers.

#### Follow-up examination in 1995

The General Health Questionnaire (GHQ) [21,22] is a measure for common mental disorders [23]. It is a widely used and well-validated self-administered test. The GHQ focuses on discontinuities in normal functioning and the experience of new phenomena of a distressing nature. It covers feelings of strain, depression, inability to cope, anxiety-based insomnia, lack of confidence and other psychological problems [24]. GHQ has been found to be very accurate at detecting anxiety and depression with anxiety [25]. We employed the 36-item version, which is derived from the original 60-item questionnaire by excluding items measuring somatic symptoms [26]. We applied the standard scoring method, counting the two highest response options as pathological. As commonly done earlier [27,28], a score 5 or higher was considered to indicate common mental disorders. Treatment contacts with mental health professionals before the follow-up examination were ascertained in 1995.

### Statistical analysis

Data were analysed with SPSS 11. Logistic regression was used to model the relationship between assumed risk factors and high GHQ score [5 or more]. Initial models included sex, social class, availability of social support [dichotomous variables], age, self-esteem, coherence of future, number of positive and that of negative life-events, neurotic, immature and mature defence styles, trait anxiety, trait depression, somatic symptom score [continuous variables]. Second-level interactions were studied by adding product terms to the models. Because of missing values, the number of cases was lower than 626 in some analyses.

Only significant [p < 0.05] confounders remained in the final models. To evaluate relative risk, fear of nuclear war and the significant confounders were categorised and odds ratios with their 95% confidence interval were estimated.

## Results

Of the 400 women, 27.5% reported having feared nuclear war once a week or more often in 1990. The respective figures for men were 226 and 13.7%. Thirty-six per cent of the women and 22.1 % of the men scored 5 or higher on GHQ. The initial full model included all putative confounders under study (Table 1). There were no interactions. Significant and almost significant explaining variables were retained in the final model with continuous variables. The risk for common mental disorders was found to be significantly related to high frequency of fear for nuclear war, high scores of trait anxiety and high scores of immature defense style (Table 2). While the odds ratios suggested a dose-response relation between fear of nuclear war and common mental disorders, significantly elevated risk was confined to the group reporting fear of nuclear war once a week or more often. This group showed a 2-fold risk compared to subjects that did not report fear of nuclear war (Table 3). High immature defense style and high trait anxiety were also related to higher risk for common mental disorders. Applying a GHQ cut-off score 6 did not materially change the results.

**Table 1:** Logistic regression analysis, General Health Questionnaire score on unit change in all potential explanatory variables (n = 607)

Explanatory variable	Odds ratio	Regression coefficient	SD	p-value
Frequency of fearing nuclear war	1.04	0.039	0.015	0.007
Sex	0.75	-0.287	0.231	0.2
Age	1.10	0.093	0.114	0.4
Social class II	1.05	0.052	0.240	0.8
Social class III	0.64	-0.439	0.263	0.095
Social class IV	1.40	0.336	0.559	0.5
Number of positive life events	1.01	0.005	0.041	0.9
Number of negative life events	1.05	0.052	0.056	0.4
Social support	1.07	0.063	0.426	0.9
Self esteem	0.97	-0.029	0.030	0.3
Coherence of future	1.00	-0.005	0.159	0.98
Trait anxiety	1.04	0.042	0.019	0.03
Trait depression	1.10	0.091	0.108	0.4
Mature defense style	0.94	-0.065	0.116	0.6
Neurotic defense style	1.10	0.096	0.120	0.4
Immature defense style	1.32	0.274	0.163	0.09
Somatic symptom score	1.02	0.021	0.025	0.4

**Table 2:** Logistic regression analysis, General Health Questionnaire score on unit change in significant explanatory variables (n = 621)

Explanatory variable	Odds ratio	Regression coefficient	SD	p-value
Frequency of fearing nuclear war	1.04	0.04	0.014	0.004
Trait anxiety	1.07	0.069	0.014	<0.001
Immature defense style	1.43	0.359	0.143	0.012

**Table 3:** Logistic regression analysis, General Health Questionnaire (GHQ) score on categorized significant explanatory variables (n = 626)

Explanatory variable		Number of cases		Odds ratio (95% confidence interval)	
		
		GHQ ≥ 5	GHQ <5	Unadjusted	Adjusted
Fear of nuclear war	never	63	199	1 (reference)	1 (reference)
	less than once a week	72	151	1.51 (1.01–2.24)	1.35 (0.88–2.05)
	once a week or more	59	82	2.27 (1.47–3.52)	2.01 (1.26–3.21)
Trait anxiety	<32	26	167	1 (reference)	1 (reference)
	≥ 32 < 38	95	195	3.13 (1.94–5.06)	2.58 (1.58–4.23)
	≥ 38	73	70	6.70 (3.95–11.4)	4.48 (2.53–7.91)
Immature defense style	<3.3	40	159	1 (reference)	1 (reference)
	≥ 3.3 < 4.0	56	148	1.50 (0.95–2.39)	1.24 (0.76–2.01)
	≥ 4.0	98	125	3.12 (2.02–4.82)	2.01 (1.24–3.25)

## Discussion

A positive association was found between frequent fear of nuclear war at baseline examination and common mental disorders among adolescents in a five-year follow up. The temporal order of exposure and response suggest that this relation could be causal. Our measure for common mental disorders, the GHQ, rates recent change (within the past month) in mental health at follow-up examination, i.e. incident problems. False positives might have included individuals with mild or transient psychological disturbance, which should have biased the association towards the null. Still, the relation was significant. However, some caveats should be discussed.

Could the association be due to some confounding factors? We controlled for several potential confounders. Those, known to increase or decrease the risk of mental disorders, included neurotic, immature and mature defence styles [29-31], trait anxiety [32,33], trait depression [34,35], life-events [36,37], somatic symptom score [38], self-esteem [39], coherence of future [40,41] and social support [42,43]. Nevertheless, on one hand there always remains the possibility of bias due to some unknown or otherwise not controlled variable, and, on the other, one cannot be sure that such a variable would also be an actual confounder in the data set at hand. In our data, the close correspondences of unadjusted and adjusted risk ratios suggest that no material residual confounding remained [44].

Adolescents not willing to answer a mental health questionnaire may have more mental health risk factors and problems than participants. However, we found no significant differences between the anonymous and identifiable respondents in possible mental health risk factors analysed except that anonymous respondents reported slightly more somatic symptoms than those who identified themselves. The difference was, however, small in absolute terms (data presented above in section on design). This suggests that subjects with high risk were not underrepresented in the present sample.

The degree of perceived threat of nuclear war may depend on several factors, such as (i) actual presence and size of the nuclear weapon arsenal, (ii) actual political tensions and threats, (iii) media coverage of the former, (iv) mental, conscious and unconscious processing of information, and (v) psychological developmental influences specific to adolescence.

Part of the fear may be based on realistic evaluation of the threat. Our baseline examination was carried out within two months before the outbreak of the Persian Gulf War in January 1991 and before the reductions in nuclear weapon arsenals in the United States and in Russia started. A quote from a novel describing the life experience of one teen-age girl during the pre-*detente *period may be illustrative:

"One was obliged to think about something important. One was obliged to think about the crisis between China and Soviet Union. A war could break out, the World War III and nuclear fallout would burn everything. The familiar fear for war pressed me inside so that it was difficult to breathe."

Laura Honkasalo. Sinun lapsesi eivät ole sinun. Jyväskylä: Gummerus, 2001, p. 132.

But similar experiences were not unknown among boys either, as witnessed by a seasoned cook from New York:

"I grew up thinking the Big One could come at any moment, and this country – or fear of it, the way my country reacted to the threat – radicalized, marginalized, and alienated me in ways that still affect me."

Alan Bourdain. A cook's tour in search of the perfect meal. London: Bloomsbury, 2001. p. 80.

Widespread media coverage on any potential danger may bring about considerable increase in perceived fear [45]. Mass media have been found to be the most important source of information about the issue of nuclear war among adolescents in Finland [5].

Perceptions of the threat of nuclear war as well as other dangers are processed mentally. Conscious or unconscious intentions are often projected to or mixed with dangerous external events, and they may distort the association between the actual threat of war and perceived fear. There is growing evidence that violent films and video games may trigger fear, aggression and violence among adolescents vulnerable to such content [46], and perceived fear of nuclear war might cause mental distress in vulnerable adolescents in similar vein.

Studies on the prevalence of fearing or worrying about nuclear war during periods of low political tension suggest that this phenomenon is common in adolescence and disappears or at least diminishes later in life [47]. Cognitive maturation and lessening of egocentrism seem to explain why fears with a major irrational component decrease from early adolescence to adulthood [48].

Global threats may vary in time as well as in their appraisal. In addition to old risks of nuclear war and aircraft hijacking, international terrorism and biological warfare loom at present. How should we handle these risks? We might inquire into the fears of our patients, appraise the risks realistically, point out that widespread media coverage tends to exaggerate the risks, and, as Durodié and Wessely [49] point out, suggest that we should not become victims of our fears.

## Conclusions

A clear positive association was found between fear of nuclear war and common mental disorders among adolescents. Fear of nuclear war may either be a risk indicator produced by an underlying vulnerability to psychopathological process or have a more direct causal role in the onset of mental disorder among adolescents. In either case, frequent fear of nuclear war in adolescence seems to be an indicator for an increased risk for common mental disorders that deserves serious attention.

## Competing interests

The authors declare that they have no competing interests.

## Authors' contributions

KP and JL planned and designed the study. KP wrote the study proposal and received funding. KP, TA-S, AT-H, MM and JL designed the follow-up, supervised the data collection, and interpreted data. KP analysed data and drafted the paper. KP revised it with contributions from all authors. All authors read and approved the final manuscript.

## Pre-publication history

The pre-publication history for this paper can be accessed here:



## References

[B1] Solantaus T, Rimpelä M, Taipale V (1984). The threat of war in the minds of 12–18-year-olds in Finland. Lancet.

[B2] Chivian E, Robinson JP, Tudge JR, Popov NP, Andreyenkov VG (1988). American and Soviet teenagers' concerns about nuclear war and the future. New Engl J Med.

[B3] Stillion JM, Goodrow H, Klingman A, Loughlin M, Morgan JD, Sandsberg S, Walton M, Warren WG (1988). Dimensions of the shadow: children of six nations respond to the nuclear threat. Death Studies.

[B4] Hamilton SB, van Mouwerik S, Oetting ER, Beauvais F, Keilin WG (1988). Nuclear war as a source of adolescent worry: relationships with age, gender, trait emotionality, and drug use. J Soc Psychol.

[B5] Nurmi JE (1988). Experience of the threat of war among Finnish adolescents: effects on thinking about the future, and comparison of methods. Med War.

[B6] Tizard B (1989). Old and new paradigms: research on young people's response to the nuclear threat. J Adolescence.

[B7] Solantaus T, Rimpelä M (1986). Mental health and the threat of nuclear war: a suitable case for treatment?. Int J Mental Health.

[B8] Thompson J The psychosocial aspects of nuclear threat and nuclear war: analogies from disaster research. In: Effects of Nuclear War on Health and Health Services. Report of the WHO Management Group on the follow-up of resolution WHA3628 Fortieth World Health Assembly, provisional agenda item 30 World Health Organization A40/11.

[B9] Poikolainen K, Kanerva R, Lönnqvist J (1994). Threat of nuclear war increases anxiety and psychosomatic symptoms among adolescents. Eur Child Adolesc Psychiatry.

[B10] Poikolainen K, Aalto-Setälä T, Marttunen M, Tuulio-Henriksson A, Lönnqvist J (2000). Predictors of somatic symptoms: a 5-year follow-up of adolescents. Arch Dis Childhood.

[B11] Bond MP, Vaillant JS (1986). An empirical study of the relationship between diagnosis and defense style. Arch Gen Psychiatry.

[B12] Andrews G, Pollock C, Stewart G (1989). The determination of defence style by questionnaire. Arch Gen Psychiatry.

[B13] American Psychiatric Association (1987). Diagnostic and Statistical Manual of Mental Disorders.

[B14] Tuulio-Henriksson A, Aalto-Setälä T, Poikolainen K, Lönnqvist J (1997). Psychological defense styles from adolescence to young adulthood: a follow-up study. J Am Acad Child Adolesc Psychiatry.

[B15] Spielberger CD, Gorsuch RL, Lushene RE (1970). STAI Manual for the State-Trait Anxiety Inventory.

[B16] Broadman K, Erdmann AJ, Wolff HG (1956). Cornell Medical Index Health Questionnaire, Manual [Revised 1956].

[B17] Johnson JH, McCutcheon SM, Sarason IG, Spielberger CD (1980). Assessing life stress in older children and adolescents: preliminary findings with the Life Events Checklist. Stress and anxiety.

[B18] Aro S (1981). Stress, morbidity, and health-related behaviour. A five-year follow-up study among metal industry employees. Scand J Soc Med.

[B19] Rosenberg M (1965). Society and the adolescent self-image.

[B20] Antonovsky A (1988). Unraveling the mystery of health: how people manage stress and stay well.

[B21] Goldberg DP (1972). The detection of psychiatric illness by questionnaire Institute of Psychiatry, Maudsley Monographs.

[B22] Goldberg DP, Gater R, Sartorius N, Ustun TB, Piccinelli M, Gureje O, Rutter C (1997). The validity of two versions of the GHQ in the WHO study of mental illness in general health care. Psychol Med.

[B23] Weich S, Lewis G (1998). Poverty, unemployment and common mental disorders: population based cohort study. BMJ.

[B24] Wall TD, Bolden RI, Borrill CS, Carter AJ, Golya DA, Hardy GE, Haynes CE, Rick JE, Shapiro DA, West MA (1997). Minor psychiatric disorder in NHS trust staff: occupational and gender differences. Br J Psychiatry.

[B25] McDowell I, Newell C (1987). Measuring health: a guide to rating scales and questionnaires.

[B26] Aalto-Setälä T, Poikolainen K, Tuulio-Henriksson A, Marttunen M, Lönnqvist J (2002). Predictors of mental distress in early adulthood: a five-year follow-up of 709 high-school students. Nord J Psychiatry.

[B27] Tarnopolsky A, Hand DJ, McLean EK, Roberts H, Wiggins RD (1979). Validity and uses of a screening questionnaire (GHQ) in the community. Br J Psychiatry.

[B28] Huppert FA, Gore M, Elliott BJ (1988). The value of an improved scoring system (CGHQ) for the general health questionnaire in a representative community sample. Psychol Med.

[B29] Bond MP, Vaillant JS (1986). An empirical study of the relationship between diagnosis and defense style. Arch Gen Psychiatry.

[B30] Pollock C, Andrews G (1989). Defense styles associated with specific anxiety disorders. Am J Psychiatry.

[B31] Soldz S, Vaillant GE (1998). A 50-year longitudinal study of defense use among innner city men: a validation of the DSM-IV defense axis. J Nerv Ment Dis.

[B32] Angst J, Vollrath M (1991). The natural history of anxiety disorders. Acta Psychiatr Scand.

[B33] Gustavsson JP, Pedersen NL, Åsberg M, Schalling D (1996). Origins of individual differences in anxiety proneness: a twin/adoption study of the anxiety-related scales from the Karolinska Scales of Personality [KSP]. Acta Psychiatr Scand.

[B34] Kandel DB, Davies M (1986). Adult sequelae of adolescent depressive symptoms. Arch Gen Psychiatry.

[B35] Gotlib IH, Lewinsohn PM, Seeley R (1995). Symptoms versus a diagnosis of depression: differences in psychosocial functioning. J Consult Clin Psychol.

[B36] Goodyer IM, Kolvin I, Gatzanis S (1987). The impact of recent undesirable life events on psychiatric disorders in childhood and adolescence. Br J Psychiatry.

[B37] Brown GW (1993). Life events and affective disorder: replications and limitations. Psychosom Med.

[B38] Kroenke K, Spitzer RL, Williams JB, Linzer M, Hahn SR, deGruy FV 3rd, Brody D (1994). Physical symptoms in primary care. Predictors of psychiatric disorders and functional impairment. Arch Fam Med.

[B39] King CA, Naylor MW, Segal HG, Evans T, Shain BN (1993). Global self-worth, specific self-perceptions of competence, and depression in adolescents. J Am Acad Child Adolesc Psychiatry.

[B40] Petrie K, Brook R (1992). Sense of coherence, self-esteem, depression and hopelessness as correlates of reattempting suicide. Br J Clin Psychol.

[B41] Suominen S, Helenius H, Blomberg H, Uutela A, Koskenvuo M (2001). Sense of coherence as a predictor of subjective state of health: results of 4 years of follow-up of adults. J Psychosom Res.

[B42] Stansfeld SA, Gallacher JE, Sharp DS, Yarnell JW (1991). Social factors and minor psychiatric disorder in middle-aged men: a validation study and a population survey. Psychol Med.

[B43] Viinamäki H, Kontula O, Niskanen L, Koskela K (1995). The association between economic and social factors and mental health in Finland. Acta Psychiatr Scand.

[B44] Greenland S, Robins JM (1986). Identifiability, exchangeability, and epidemiological confounding. Int J Epidemiol.

[B45] Slovic P (1987). Perception of risk. Science.

[B46] Villani S (2001). Impact of media on children and adolescents: a 10-year review of the research. J Am Acad Child Adolesc Psychiatry.

[B47] Gleisner JW (1986). The threat of nuclear war: a study of some of the psychological effects on adults. Med War.

[B48] Elkind D (1967). Egocentrism in adolescence. Child Dev.

[B49] Durodié B, Wessely S (2002). Resilience or panic? The public and terrorist attack. Lancet.

